# New Alternatives to Milk From Pulses: Chickpea and Lupin Beverages With Improved Digestibility and Potential Bioactivities for Human Health

**DOI:** 10.3389/fnut.2022.852907

**Published:** 2022-07-14

**Authors:** Carla Margarida Duarte, Joana Mota, Ricardo Assunção, Carla Martins, Ana Cristina Ribeiro, Ana Lima, Anabela Raymundo, Maria Cristiana Nunes, Ricardo Boavida Ferreira, Isabel Sousa

**Affiliations:** ^1^LEAF-Linking Landscape, Environment, Agriculture and Food, Instituto Superior de Agronomia, University of Lisbon, Lisbon, Portugal; ^2^IUEM, Instituto Universitário Egas Moniz, Egas Moniz-Cooperativa de Ensino Superior, CRL, Costa da Caparica, Portugal; ^3^Department of Food and Nutrition, National Institute of Health Doutor Ricardo Jorge, Lisbon, Portugal; ^4^CESAM, Centre for Environmental and Marine Studies, University of Aveiro, Aveiro, Portugal; ^5^NOVA National School of Public Health, Public Health Research Center, Universidade NOVA de Lisboa, Lisbon, Portugal; ^6^Faculdade de Farmácia de Lisboa, University of Lisbon, Lisbon, Portugal; ^7^Faculty of Veterinary Medicine, Lusófona University, Lisbon, Portugal

**Keywords:** non-dairy beverages, pulses, chickpea, lupin, digestibility, bioaccessibility, bioactivity

## Abstract

There is a strong demand for plant-based milk substitutes, often low in protein content (<1.5% w/v). Protein-rich pulse seeds and the right processing technologies make it possible to make relevant choices. The major objective of this study was to assess the impact of processing on the nutritional characteristics of beverages with a high impact on health, in particular on digestibility and specific bioactivities. The results suggest that pulse beverages are as high in protein content (3.24% w/v for chickpea and 4.05% w/v for lupin) as cow’s milk. The anti-nutrient level characteristics of pulses have been considerably reduced by strategic processing. However, when present in small quantities, some of these anti-nutritional factors may have health benefits. Controlling processing conditions play a crucial role in this fine balance as a tool to take advantage of their health benefits. There is evidence of protein hydrolysis by *in vitro* digestion and limited bioaccessibility of minerals. In addition to being highly digestible, lupin and chickpea beverages have anti-inflammatory and anti-carcinogenic potential evaluated through the inhibition of metalloproteinase MMP-9.

## Graphical Abstract

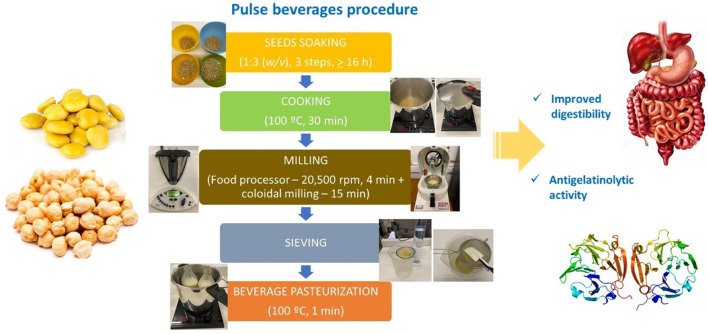

## Introduction

Plant-based beverages represent a major consumer trend and a fast-growing segment of the food market. Currently, the dairy alternative global market was projected to grow from USD$17.3 billion in 2018 to USD$29.6 billion by 2023, at a compound annual growth rate (CAGR) of 11.4%, with the Asia-Pacific region representing the biggest market share (1, 2). There is a strong demand for milk alternatives other than soy beverages for different reasons, ranging from health-related issues (e.g., lactose intolerance, milk protein allergies, hypercholesterolemia, presence of hormones, antibiotics, and pesticide residues), sustainability and ethical grounds, such as concerns relating livestock, to huge environmental impacts (3–6), representing 14.5% of all human-induced CO_2_ emissions (7), as well as crops related to deforestation and long-run transportation, such as soy. However, current market offers are essentially poor in protein, except soy beverages (3–4% w/v) and cereal and nut-based beverages, such as those made from rice, oat, or almonds containing between 0.1 and 1.5% protein (w/v) (8) compared to 3.3–3.5% (w/v) of protein in cow’s milk. Therefore, developing protein-rich drinks from pulses, such as chickpea and lupin, can be proposed as an effective alternative to cow’s milk.

Pulses are well known for their nutritional and health-promoting properties, and for being sustainable crops that incorporate nitrogen in the soil, significantly reducing fertilizer use. Pulses are a good source of protein, minerals, and fiber, and their beverages have a good nutritional composition with a low glycemic index (9, 10). Contrary to sustainability and fair production, a major issue hampers the development and widespread production of legume beverages: the “beany flavor,” which is so negatively popular and notorious in soy beverages. This “raw” or beany flavor is associated with the activity of lipoxygenases and is strengthened in the presence of polyphenols (bitter taste). This is typically related to soy beverages since soy is high in fat (20% dry matter), while chickpeas and lupins are low in fat (1–5%). The technique used to remove off-flavors from legume-based beverages is thermal inactivation, but high temperatures lead to denaturation, aggregation, reduction in protein solubility, and nutrient losses (e.g., vitamins and minerals), which have restricted its use as a promising technology. In addition, some bioactive compounds from legume seeds, such as protease inhibitors (PIs), seed reserve proteins (γ-conglutin), lectins, phytates, oligosaccharides, saponins, and phenolic compounds may have important metabolic effects on the consumer’s health (11–15). Some of these substances are beneficial, while others are considered anti-nutritional factors, which overall impair digestion, such as PIs, which decrease the digestibility and absorption of proteins (16). Phytates bind microelements by reducing their availability and do not degrade during cooking (16). So, their overall effective reduction can only be achieved by enzymatic degradation, chelation, germination and fermentation (5, 17), or intensive soaking as they are fairly soluble in water.

Some anti-nutritional factors and their breakdown products may have beneficial health effects if they are present in small quantities. Manipulating the processing conditions, in addition to removing certain unwanted compounds from foods, may be required to eliminate the deleterious effects of anti-nutritional factors and take advantage of their health benefits (18). Several studies have shown the positive impact of these substances, namely on the prevention of diabetes (13), as well as on cancer prevention and therapy (19). Concerning the latter example, research with animal models have shown that dietary Bowman-Birk inhibitors (BBIs) from several legume sources, including soybean, pea, lentil, and chickpea, can prevent or suppress inflammatory and carcinogenic processes within the gastrointestinal tract (20, 21). Other *in vitro* and *in vivo* studies showed that the antioxidant properties of phytic acid have a beneficial role in cancer prevention and a positive effect on the growth inhibition of several types of cancer (22). Recent studies have shown that protein extracts from some legume seeds, particularly the albumins from *Lupinus albus*, can inhibit the metalloproteinase MMP-9 involved in inflammation and cancer, as well as colon cancer cell migration (23, 24). Furthermore, heat-resistant Pis in soy have shown to be more effective and selective against MMP-9 than non-proteins compounds (e.g., isoflavones, saponins) (25). Because of its important roles in inflammatory and cancer diseases, MMP-9 is an important disease biomarker and clinical target, and its inhibitors are currently regarded as important alternatives to deal with various human ailments, such as inflammatory bowel diseases (IBD), cancer, cardiovascular diseases, osteoporosis, and even neurological disorders (26). In fact, several works (23, 27, 28) have shown that some poorly studied legume seeds, such as lupin and chickpea, have higher bioactivities than others, such as soybean, and are very effective in reducing cancer metastasis and colitis *in vivo*.

Under this context, the present study aimed at evaluating the impact of processing in maintaining the nutritional characteristics of chickpea and lupin-based beverages while striving to achieve their best digestibility, as well as protein and mineral bioaccessibility. The maintenance of selected bioactivities present in these two pulses was also pursued, both during processing and digestion. Overall, the current study revealed that the two pulse-based beverages may provide an effective alternative to cow’s milk protein and other less nutritional and digestible plant-based alternatives, with further added benefits to human health.

## Materials and Methods

### Pulse Beverage Production

The experimental work used two different pulse seeds, sweet lupin (*Lupinus albus* L.) and chickpea (*Cicer arietinum* L.), to produce beverages containing 10% (w/v) of whole dry seeds, without peeling. Two batches per pulse beverage were produced and this procedure was repeated three times to verify the repeatability. Briefly, 150 g of dried seeds, previously soaked in water (1:3 w/v) for 16 h, changing the water two times in the same proportion, were cooked for 30 min in a pressure cooker (7.5 L, Mod. Qualix 8, Magefesa) into 1.5 L of tap water. Cooked pulse seeds were drained and 1.5 L of fresh tap water was added. The milling step included the food processor at 20,500 rpm (Bimby Worwerk, Wuppertal, Germany) for 4 min, followed by colloid milling simulated by a mortar grinder (Pulverisette 2, Fritsch GmbH, Germany) at a lab scale at 70 rpm for 45 min, at room temperature. Both chickpea and lupin beverages were sieved with a strainer, and their particle diameters were marked from an analysis carried out in a previous study (8). Finally, the pulse beverages were poured into sterilized bottles and were kept in a water bath inside the pressure cooker. When the temperature of the beverage reached a minimum of 90°C, the bottles were tightly capped and kept inside the pressure cooker closed for 1 min.

### Digestibility Tests

Chickpea and lupin beverages were subjected to the static *in vitro* digestion method (29) comprising three sequential simulated digestive phases: oral, gastric, and intestinal. This procedure was performed three times to verify the repeatability in protein and mineral bioaccessibility values. Briefly, 2 mL of each beverage was diluted 1:1 (v/v) in simulated salivary fluid (SSF) with alpha-amylase from *Bacillus* sp. (75 UI/mL) and incubated for 2 min at 37°C under continuous agitation in an overhead rotator (Reax 2, model 444-1113, Heidolph Instruments, Germany). The volume of simulated gastric fluid (SGF) with porcine pepsin (2,000 UI/mL) to dilute the oral bolus was 1:1 (v/v) and the pH was adjusted to 3 with 10 M HCl, when necessary. The mixture was incubated at 37°C with agitation for 2 h. The gastric chyme was diluted 1:1 (v/v) with simulated intestinal fluid (SIF), and the bile salts (10 mM) and pancreatin (100 UI/mL of trypsin activity) were added followed by adjustment to pH 7 (with 1 M NaOH). Incubation at 37°C under agitation was stopped after 2 h with Pefabloc, a serine PI (5 mM). An enzyme-blank tube was included in these trials where the 2 mL of pulse beverage was replaced by demineralized water. To obtain the soluble (potentially bioavailable) and insoluble fractions after *in vitro* digestion, the whole *digesta* was centrifuged at 6,000 × *g* for 10 min, at 4°C.

### Bioaccessibility Calculations

The term bioaccessibility refers to the fraction of protein and mineral elements that was released from the pulse beverages during *in vitro* digestion and that presumably becomes accessible (available) for absorption through the small intestine walls. Bioaccessibility should be distinguished from the term bioavailability, which is defined as the fraction of nutrients or food components that have been efficiently digested *in vivo*, assimilated, and then absorbed in the body (30). Therefore, one could say that the bioaccessibility of the study nutrients is a prerequisite for their bioavailability. The bioaccessibility of protein and minerals (31) was calculated using triplicates as:


(1) [Bioaccessibility=DB⁢x⁢ 100]

where *D* is the concentration of the soluble nutrient in the *digesta*, corrected with the correspondent nutrient content in the enzyme-blank, and *B* is the initial concentration of the same nutrient in the beverage, before digestion.

### Gel Electrophoresis for Protein Hydrolysis Analysis

The protein and polypeptide patterns before and after *in vitro* digestion were analyzed two times by reducing sodium dodecyl sulfate–polyacrylamide gel electrophoresis (SDS PAGE) in 17.5% (w/v) polyacrylamide gel (32) and stained by silver nitrate (33).

Volumes corresponding to 15 μg of protein were considered for chickpea and lupin beverages, and 7 μg were considered for their respective *digesta*. The enzyme-blank has also been used with a protein content of 7 μg to compare the protein profiles of enzyme addition to the whole *digesta*. All samples were previously diluted at a ratio of 1:5 in a 100 mM Tris HCl buffer containing 0.3 M NaCl to promote protein solubilization.

### Physico-Chemical Analysis

The total protein determination was performed by Dumas Nitrogen Analyser NDA 702 (Velp Scientifica) and the correction factor used to convert nitrogen to crude protein was 5.4 (34). All analyses were carried out in triplicate and expressed in grams of protein per 100 mL of sample (or %w/v).

The total starch analysis was determined according to the Megazyme Total Starch Assay Procedure (K-TSTA) based on the AOAC official method 996.11 (2005), regarding specific procedures for samples in which the starch is present in a soluble or suspended form. The analyses were carried out in duplicate and expressed in grams of starch per 100 mL of sample.

The D-glucose content was obtained by high-performance liquid chromatography (HPLC) (35). Briefly, 2 mL of each sample was centrifuged at 7,200 × *g* for 10 min and 500 μL of supernatant was collected. After its dilution in H_2_SO_4_ (50 mM) (1:1 v/v), the samples were centrifuged (7,200 × *g*, 10 min) to discard the precipitated protein and filtered under vacuum through a 0.20 μm-pore-size filter. Glucose was quantified in an HPLC system (Waters Alliance, Milford, MA, United States) equipped with a refractive index detector (Waters 2414) and a Rezex™ ROA Organic Acid H+ (8%) column (300 mm × 7.8 mm, Phenomenex, Torrance, CA, United States), at 65°C. Sulfuric acid (5 mM) was used as the mobile phase at 0.5 mL min^–1^. The analysis was performed in triplicate and the results are expressed in grams of glucose per 100 mL of sample.

Total carbohydrate content was carried out in triplicates and according to the Dubois’ method (36). The results are expressed in grams of total carbohydrates per 100 mL of sample.

The ash content was determined gravimetrically by incineration of triplicates at 550°C in a muffle furnace (Snol LHM01, Utena, Lithuania) using the AOAC 923.03 method (37). The results are given in grams of ash per 100 mL of sample.

Dry matter was also determined gravimetrically by drying at 105°C in a forced-air oven (Binder, Germany) until the constant weight of the sample according to the AOAC 934.01 method (38), and solid residue in beverages was calculated as a percentage for triplicates.

The energy value of each pulse beverage was calculated considering the conversion factors (39) for protein (4 kCal/g; 17 kJ/g), fat (9 kCal/g; 37 kJ/g), and carbohydrates (4 kCal/g; 17 kJ/g). The calculation of fat content for each pulse beverage took into account that 10 g of dry pulse seed (in 100 mL) contained 0.50 g of fat for chickpea and 0.24 g of fat for lupin (40).

Minerals’ profile was evaluated by inductively coupled plasma optical emission spectrometry (ICP-OES: iCAP 7000 Series Spectrometer equipped with ASX-520 AutoSampler, Thermo Scientific, Waltham, MA, United States) based on the AOAC 984.27 method (41). Briefly, 5 mL of each sample was transferred into digestion vessels and 9 mL of HCl (37%) and 3 mL of HNO_3_ (65%) were added. The digestion (SCP Science, DigiPREP MS, Baie d’Urfe, QC, Canada) occurred at 15 min/45°C, 15 min/80°C, and 60 min/105°C. After cooling, up to 50 mL of distilled water was added and the solution was left to settle. Finally, the clear supernatant was used for the ICP analysis. Eleven elements (Na, K, Ca, Mg, P, S, Fe, Cu, Zn, Mn, and B) were identified in triplicate. Results are expressed in milligrams of mineral element per 100 mL of sample.

### Anti-nutrient Analysis

The phytic acid content of the beverages was determined in triplicate using the Gao et al.’s method (42). Briefly, the freeze-dried samples were kept under agitation at 220 rpm for 16 h in HCl 2.4% (v/v) (1:20 w/v). Further centrifugation of the samples was performed at 1,000 × *g* for 20 min at 10°C in a Beckman Coulter™ Allegra™ 25R centrifuge until clear supernatants were obtained. Supernatants were collected for color development. A calibration curve was previously obtained from a 25 mg/mL phytic acid solution (SC-250718, Santa Cruz Biotechnology, TX, United States) varying from 0 to 3.5 mg/mL. The modified Wade reagent [0.03% (w/v) FeCl_3_⋅6H_2_O + 0.3% sulfosalicylic acid (w/v)] was added to volume samples in a proportion of 3:1 (v/v), thoroughly mixed on a vortex, and centrifuged at 1,000 × g during 10 min at 10°C in a centrifuge Himac CT15RE. The absorbance was measured at 500 nm using the Synergy HT spectrophotometer, Bio-TEK (Agilent, CA, United States). Final results are expressed in grams of phytic acid per 100 mL of beverage and in mg/g of phytic acid per weight of the beverage.

Lectins activity (hemagglutination activity) of sample protein extracts was performed according to the method described by Ribeiro et al. (43). Briefly, both chickpea and lupin beverages, as well as their *digesta*, were first desalted through PD-10 columns previously equilibrated in saline solutions (0.9% w/v NaCl), following ultrafiltration at 1,400 × *g* and 4°C (Macrosep MWCO: 10kD, Pall Corporation, NY, United States) of all samples, in a way to wash them with saline and reduced to volumes containing 50 and 100 μg of protein. The last centrifugation was carried out with a saline solution containing 2 mM CaCl_2_ and 2 mM MgCl_2_. For the hemagglutination activity, 5 mL of rabbit erythrocytes was used and washed three times in a saline solution by centrifugation (1,800 × *g*, 12°C, 6 min), after which they were incubated with trypsin (T0303, Sigma-Aldrich, Merck, Darmstadt, Germany) and shaken at 120 rpm for 1 h at 37°C, at a final concentration of 0.1% (w/v) in a saline solution. The suspension of 4% (v/v) of trypsinized erythrocytes was stored at 4°C and used for the hemagglutination activity assays. The measurements of hemagglutination activity required the protein analysis (50–100 μg in 50–70 μL saline) through serially dilution (1:2) in a 96-well microplate. The erythrocyte suspension (50–70 μL) was then added and the microplate was incubated for 30 min at 37°C before visual evaluation. Both positive [Con-A lectin (concanavalin A) at 0.5 mg/mL] and negative (saline) controls were prepared. One H.U. (Hemagglutination Unit) is defined as the minimal protein concentration, which induces erythrocyte agglutination (*n* refers to dilution).


$$H.U.=\frac{\mathrm{Total}\,\mathrm{protein}\,\mathrm{concentration}}{2^{n-1}}$$

### *In vitro* Assays With HT-29 Cells

#### HT29 Cell Cultures

The human colon adenocarcinoma cell line, HT29 (ECACC 85061109), obtained from a 44-year-old Caucasian female, was used. HT29 cells were maintained according to Lima et al. (23).

#### Wound Healing Assay

For cell migration analysis, the wound healing assay was performed according to Lima and coworkers (23). HT29 cells (5 × 10^5^ cells/well) were seeded in 24-well plates and allowed to reach 80% confluence. Each well was subsequently supplemented with a fresh medium containing the water-soluble protein extracts of both beverages at a concentration of 100 μg mL^–1^. The invaded area after 48 h was calculated for each treatment and compared to the initial area at 0 h.

#### Cell Proliferation and Viability Assays

HT29 cultured cells were seeded in 96-well plates (2 × 10^4^ cells/well), and the soluble protein extracts of both beverages were added at a concentration of 100 μg mL^–1^ as described above, and the cells were incubated for 48 h. The extracellular medium was then collected, the wells were washed with phosphate-buffered saline (PBS) to remove the unattached cells, and the cell growth and viability were determined using the MTT (3-[4,5-dimethylthiazole-2-yl]-2,5-diphenyltetrazolium bromide) assay as described in an earlier study (23).

#### MMP-9 Catalytic Activity in HT-29 Cells

MMP-9 gelatinolytic activities in the culture media after exposure to both beverages for 48 h were determined using the DQ (dye-quenched) gelatin assay, as described by Lima et al. (23).

### Antigelatinolytic Bioactivity of Both Beverages and Respective Digesta

Lupin and chickpea beverages as well as their respective *digesta* soluble fractions were then subjected to the DQ gelatinolytic activity assay to test their inhibitory activity upon MMP-9. Briefly, the fluorogenic substrate DQ-gelatin was acquired from Invitrogen (Carlsbad, CA, United States) and dissolved in water at 1 mg/mL. All solutions and dilutions were prepared in assay buffer (50 mM Tris–HCl buffer, pH 7.6, containing 150 mM NaCl, 5 mM CaCl_2_ and 0.01% v/v Tween 20). A 96-well micro-assay plate (chimney, 96-well, black) was used. Each well was loaded with 0.1 mM (for a final volume of 200 μL) MMP-9 (Sigma), to which 100 μg/mL of total protein fraction from each legume beverage and also their *digesta* (for a final volume of 200 μL) was added, and the plate was incubated for 1 h at 37°C. Subsequently, DQ-gelatin (at a final concentration of 2.5 μg/mL) was added to each well and the plate was left to incubate again for 1 h. Fluorescence levels were measured (ex. 485 nm/em. 530 nm). In each experiment, positive (no protein fraction) and negative (no enzyme) controls were included for all samples to correct for possible proteolytic activities present in the protein samples. All data were corrected by subtracting the corresponding negative controls. Triplicates were used for each sample.

### Experimental Design

A complete randomized design (CRD) was used due to low variability in beverage production and digestion (two factors). The two pulse beverages were subjected to thirteen different analysis procedures (13 levels), resulting in nine physicochemical results, bioaccessibility value, inhibitory activity on MMP-9, and evaluation of cancer cell migration and proliferation. The two respective *digesta* were also tested for lectin activity, mineral content, bioaccessibility estimation, and inhibitory activity on MMP-9 (four levels). The replication number has at least 3.

### Statistical Analysis

All statistical processing was carried out using SPSS Statistics (v.20, IBM SPSS Statistics, New York, NY, United States). An analysis of variance (one-way ANOVA) was used to assess significant differences between samples at a significance level of 95% (*p* < 0.05). Multiple comparisons were performed by the Tukey HSD test.

## Results

### Nutritional Composition

The nutritional profile of pulse beverages is shown in Table 1. The energy contribution of protein, carbohydrates, and fat is 24.2, 67.4, and 8.4% for chickpea beverages, and 51.5, 41.6, and 6.9% for lupin beverages, respectively.

**TABLE 1:** Comparison of the nutritional composition of beverages.

Beverage	Energy (kcal/100 mL) (kJ/100 mL)	Protein (g/100 mL)	Total carbohydrates (g/100 mL)	Starch (g/100 mL)	Glucose (g/100 mL)	Fat* (g/100 mL)	Ash (g/100 mL)	Moisture (%)
Chickpea	53.49^a^ + 1.57 (226.70 + 6.67)	3.24^b^ + 0.16	9.01^a^ + 0.34	1.39^a^ + 0.22	0.06^b^ + 0.00	0.50	0.16^b^ + 0.01	93.94^a^ + 0.15
Lupin	31.44^b^ + 1.96 (133.32 + 8.31)	4.05^a^ + 0.25	3.27^b^ + 0.65	0.01^b^ + 0.06	0.09^a^ + 0.00	0.24	0.20^a^ + 0.02	93.78^a^ + 0.02

*Values are represented as mean ± standard deviation (SD) (n = 3). Distinct letters in samples per parameter, evidence significant differences among them (p < 0.05).*

*Calculation as explained in section “Physico-Chemical Analysis”.

Evaluation of the nutritional composition revealed that lupin and chickpea beverages are important sources of protein. Lupin beverage had a higher (4.05% w/v) significant value (*p* < 0.05) than chickpea beverage (3.24% w/v).

The significant higher starch content of the chickpea beverage (1.391 g/100 mL), when compared to the lupin beverage (0.008 g/100 mL), was already expected from the predictable starch content of these pulses: 10 g of dried seeds (in 100 mL) contain 4.5 g of starch in chickpea and 0.7 g in lupin (40). The values obtained in beverages confirmed the release of starch from seeds during processing/cooking and are compatible with their carbohydrate composition and contents (also significantly higher in chickpea beverage). Adding to that, the chickpea beverage did not contain resistant starch and showed starch hydrolysis into glucose following the addition of α-amylase during beverage production (data not shown) to overcome its high viscosity/consistency caused by starch gelatinization [as observed in our previous work (8)].

The lupin beverage showed a significantly higher glucose content (*p* < 0.05) than the chickpea beverage (Table 1). *Lupinus albus* does not store glucose as starch in seeds, like chickpea, but is naturally higher in glucose-containing oligosaccharides (44, 45).

The low ash content in both beverages was expected as they contained only 10% (w/v) of dry seed.

### Anti-nutrients Analysis

Regarding phytic acid, this anti-nutrient can form a complex with proteins, altering their structure, and making them less soluble, and this affects enzymatic degradation and peptic digestion (46, 47). Despite that, the results showed that the phytic acid content in both pulse beverages (Table 2) did neither inhibit the digestive enzymes during static digestion nor impair protein digestibility. This was confirmed by the good bioaccessibility of the peptides produced during the *in vitro* digestion of both beverages (Table 3).

**TABLE 2:** Phytic acid content of pulse-based beverages.

Beverage	Phytic acid (g/100 mL)
Chickpea	0.78^b^ + 0.01
Lupin	0.83^a^ + 0.00

*Values are represented as mean ± SD (n = 3). Different letters in samples evidence significant differences between them (p < 0.05).*

**TABLE 3:** Protein content before and after *in vitro* digestion in chickpea and lupin beverages.

	Beverage protein content (%)	Soluble protein after digestion (%)	Bioaccessibility (%)
Chickpea	3.24^a^ + 0.16	3.30^a^ + 0.07	102
Lupin	4.05^b^ + 0.25	3.88^b^ + 0.00	96

*Bioaccessibility percentages are shown for each pair of beverage–digesta. Values are represented as mean ± SD (n = 3). Same letters in protein content per sample evidence no significant differences between parameters (p ≥ 0.05).*

Phytic acid can also block the absorption of minerals from foods, interfering with nutrient bioavailability by affecting the intestinal absorption of some important essential minerals that are needed to meet the requirements of calcium, magnesium, iron, and zinc (48) in the human body. Still, the existing phytic acid contents in beverages (Table 2) did not evidence interference in mineral digestion as divalent cations, such as Ca, Mg, and Fe, showed some bioaccessibility after *in vitro* digestion (Table 4). The heating effect of the cooking step was more efficient to soften the vegetable tissue of chickpea seeds than lupin, allowing a higher permeability of minerals and phytic acid into the cooking water, being lost. This may explain the relevant bioaccessibility of divalent cations in the lupin beverage and its slightly higher concentration (0.83 against 0.78 g/100 mL) of phytic acid when compared to the chickpea beverage.

**TABLE 4:** Mineral content before and after *in vitro* digestion of chickpea and lupin beverages.

Mineral content								
	Chickpea beverage (mg/100 mL)	% DRI	Soluble fraction after digestion (mg/100 mL)	Bioaccessibility (%)	Lupin beverage (mg/100 mL)	% DRI	Soluble fraction after digestion (mg/100 mL)	Bioaccessibility (%)
Na	14.20^b^ + 1.55	0.95	_	_	17.62^a^ + 0.97	1.17	_	_
K	35.23^b^ + 2.53	1.76	_	_	42.10^a^ + 1.92	2.10	2.41 + 1.09	5.73
Ca	15.27^b^ + 0.66	1.91	_	_	23.62^a^ + 0.80	2.95	0.69 + 0.19	2.93
Mg	7.94^b^ + 0.48	2.15	0.33 + 0.01	4.15	10.64^a^ + 0.64	2.87	1.32 + 0.18	12.43
P	19.89^b^ + 0.72	2.84	_	_	37.32^a^ + 1.68	5.33	5.35 + 0.55	14.33
S	13.92^b^ + 0.72	_	_	_	19.28^a^ + 0.26	_	1.86 + 1.07	9.65
Fe	0.36^b^ + 0.01	2.79	_	_	0.77^a^ + 0.01	5.94	0.24 + 0.00	31.43
Cu	0.06^a^ + 0.00	6.50	_	_	0.05^a^ + 0.00	6.05	0.005 + 0.002	8.57
Zn	0.17^b^ + 0.01	1.82	_	_	0.33^a^ + 0.00	3.50	_	_
Mn	0.19^b^ + 0.01	9.03	0.01 + 0.00	2.94	3.36^a^ + 0.17	163.68	0.19 + 0.00	5.64
B	0.03^b^ + 0.00	0.13	_	_	0.06^a^ + 0.00	0.32	_	_

*Bioaccessibility percentages are shown for each pair of beverage–digesta. The mineral contribution of 100 mL of pulse beverages, taking into account the dietary reference intakes (DRI) for adults (39, 57), is also presented as a percentage. Values are represented as mean ± SD (n = 3). Different letters per mineral element represent a significant difference between pulse beverages (p < 0.05).*

**FIGURE 1:**
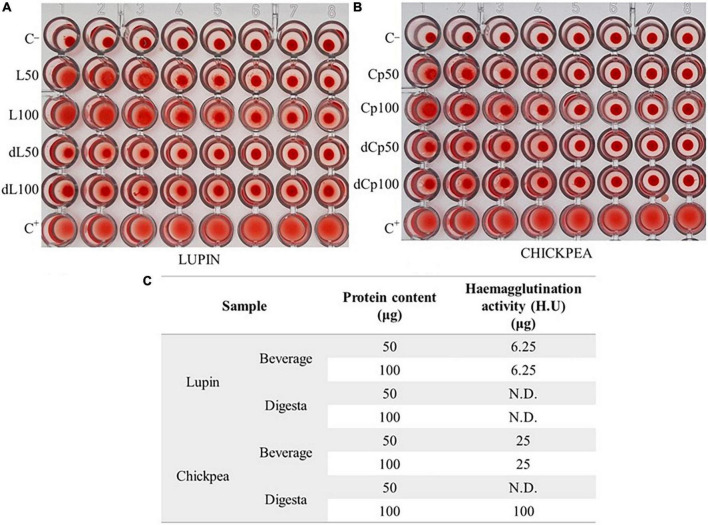
Hemagglutination activity **(A,B)**: serial dilutions (1:2) of the beverage and *digesta* extracts (50 or 100 μg of protein; L, lupin; dL, lupin *digesta*; Cp, chickpea; dCp, chickpea *digesta*) and negative (C–; saline) and positive (C+; 35 μg Con-A) controls. **(C)** Hemagglutination activity H.U. (μg) of assayed samples.

Concerning the evaluation of the lectin activity in beverages (Figure 1), assessed by the hemagglutination assay, it was shown that lupin beverage had a higher activity (U.H. 6.25 μg), despite the protein concentration, when compared to the respective *digesta* without lectin activity (Figures 1A,C) and to the chickpea beverage (U.H. 25 μg) (Figures 1B,C). Concerning chickpea, its *digesta* revealed no activity for the 50 μg protein sample tested, and an H.U. of 100 μg for the higher protein content tested, meaning a very low-lectin activity.

### Protein and Mineral Bioaccessibility

Chickpea and lupin beverages showed very good protein digestibility, resulting in bioaccessibility values of 102 and 96%, respectively (Table 3). Bioaccessibility values above 100% may result from an experimental error related to the protein quantification methodology. Nevertheless, there was no significant difference between the protein content of beverages and the corresponding soluble *digesta* fractions. The results indicated that the pulse beverages in this study contained completely digested soluble peptides. These are promising results on protein availability for absorption after digestion of pulse beverages.

The lupin beverage showed the highest mineral content compared to the chickpea beverage, and it was significant (*p* < 0.05) for the majority of the elements (Table 4). Concerning the soluble (bioavailable) fractions obtained from *in vitro* digestion, the major mineral elements of chickpea beverage were not bioaccessible, except for magnesium and manganese with only 4.15 and 2.94%, respectively. On the contrary, lupin beverage presented bioaccessibility values for almost all minerals, out of which, iron was 31.43%, phosphorous was 14.33% and magnesium was 12.43%. These values can be explained by the intrinsic mineral richness of lupin when compared to the chickpea seeds used.

### Protein Hydrolysis Analysis

**FIGURE 2:**
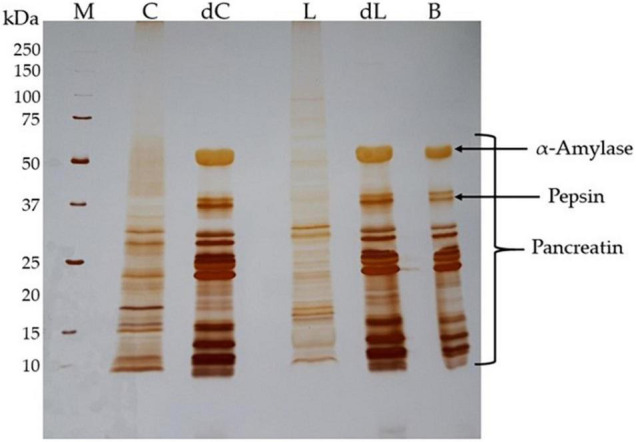
Protein hydrolysis after *in vitro* digestion. Silver-stained SDS-PAGE gel (17.5% w/v polyacrylamide) ran under reducing conditions. Expected molecular weights are indicated for the molecular weight standards. The quantity of protein loaded in each lane is 15 μg for chickpea and lupin beverages, and 7 μg for both *digesta* and enzyme-blank control. M, protein molecular weight markers; C, chickpea beverage polypeptides; dC, chickpea whole *digesta* polypeptides; L, lupin beverage polypeptides; dL, lupin whole *digesta* polypeptides; B, enzyme-blank control.

Protein digestion by the static *in vitro* method (29) has also been shown by electrophoresis (Figure 2). Polypeptide profile differences between the beverage and the respective whole *digesta* are evident in the gel stained by the silver nitrate method, which has a sensitivity to reveal low-abundance molecular weight polypeptides. The lupin beverage (L) presented a broad polypeptide profile with more representative molecular weights than the chickpea beverage (C), while *digesta* showed polypeptides with molecular weights under 50 kDa mixed with the commercial enzymes added. Comparing the differences between *digesta* and the enzyme-blank profiles, the *digesta* exhibited some low molecular weight peptides (<10 kDa) and several polypeptides under 25 kDa that are distinct from the blank (B). This revealed the high digestibility suffered in the *digesta* as a result of the enzymes used.

The electrophoretic profile of the enzyme control (B) has a match with the beverage *digesta*, evidencing all the enzymes used during the protocol, as expected. The α-amylase used showed a molecular weight of around 58–62 kDa, the pepsin of 36 kDa, and pancreatin comprises a group of several enzymes with molecular weights from 13 to 64 kDa (49) (Figure 2).

### Bioactivities

To understand if beverage processing conditions kept the anti-inflammatory and anti-cancer potential of both pulse seeds, particularly in relation to their inhibitory ability on gelatinase MMP-9, a matrix metalloproteinase, which is related to inflammation and cancer disease, we set out to test the *in vitro* inhibitory activity of the soluble protein fractions of both beverages on MMP-9, as well as their potential against colon cancer cell proliferation and migration, using an HT-29 cultured cell line. Table 5 shows the inhibitory activity of protein extracts from both beverages (100 μg mL^–1^) on MMP-9 activity, cancer HT-29 cell migration, using the wound healing assay, and cancer HT-29 cell proliferation, using the MTT assay.

**TABLE 5:** Effect of protein extracts from chickpea and lupin-based beverages on MMP-9 activity and on the migration and proliferation of HT-29 cells.

	MMP-9 activity (% of controls)	Cell migration (% of controls)	Cell proliferation (% of controls)
Chickpea beverage	23.01 ± 0.43^a^	71.94 ± 17.37^a^	89.33 ± 10.87
Lupin beverage	12.22 ± 1.21^a^	43.35 ± 16.86^b^	82.84 ± 4.91

*Cells were treated with protein extracts (100 μg mL^–1^) from each beverage. The remaining MMP-9 activity was evaluated using the DQ-gelatin assay. Cell migration was evaluated using the wound-healing assay and cell proliferation was assessed using the MTT assay. Values are presented as a percentage of controls and expressed as means ± SD (n = 3). Different letters represent significance at p < 0.05 vs. control.*

The results show that both pulse-based beverages were able to significantly (*p* < 0.05) reduce MMP-9 activity and cancer cell migration of HT-29 cells, while not reducing HT-29 cell proliferation in a significant manner. Although both beverages present similar results on MMP-9 activity and cell proliferation, the reduction in cell migration was significantly higher with the lupin beverage when compared to the chickpea beverage (*p* < 0.05).

**FIGURE 3:**
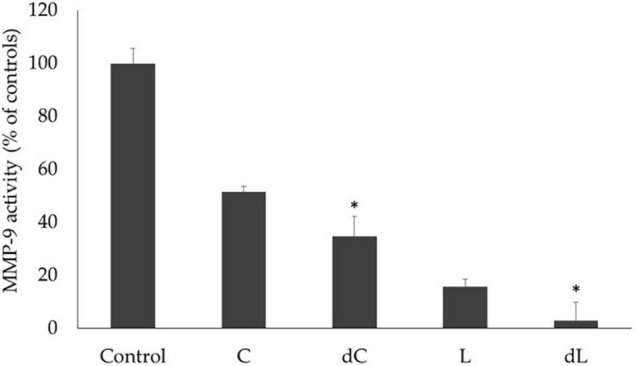
The effect of the beverages and *digesta* extracts (L, lupin; dL, lupin *digesta*; C, chickpea; dC, chickpea *digesta*) on MMP-9 gelatinolytic activity was measured by the DQ-gelatin assay. MMP-9 activity is expressed as relative fluorescence as a % of control and corresponds to the means of at least three replicate assays (*n* = 3) ± SD. *p < 0.05.

We further evaluated if the MMP-9 inhibitory activity of both beverages was also maintained after digestion by comparing the effect of both beverages and their respective *digesta* in the activity of commercial MMP-9 using the standard DQ-gelatin assay. The results are shown in Figure 3.

Overall, both beverages presented very significant inhibitory activities on commercial MMP-9 (*p* < 0.001), which were considerably higher after *in vitro* digestion, particularly for the lupin beverage, with a 96% reduction in the MMP-9 activity, as opposed to the chickpea beverage, for which a 48% inhibition was obtained (*p* < 0.05).

## Discussion

The pulse seeds chosen for new vegetable beverages, such as chickpea and lupin, led to rich protein contents similar to that of cow milk, being good nondairy alternatives. Only the protein from soy beverages, with the exception of low amounts of cysteine and methionine, was comparable to cow’s milk when nutritional composition and health benefits are discussed among commercial vegetable alternatives (50). Several advantages for human health are attributed to chickpea proteins and lupin proteins: the lupin lectin γ-conglutin, a globulin composed of 42 kDa subunits, showed beneficial properties through its reducing effect in glycemia being suitable for diabetics (14, 51); the deflamin protein found in legume seeds, namely lupin and chickpea, has been shown to be anti-inflammatory in *in vitro* models with various colon cancer cell lines as well as *in vivo* models of acute and chronic disease, presenting inhibitory bioactivity against MMP-9 (24, 27). Also, the seed reserve protein β-conglutin from *Lupinus albus* exerted action on glycemic modulation and reduced the circulating cholesterol (15), and a natural 210 kDa glyco-oligomer fungicide, termed Blad-containing oligomer present in *Lupinus albus*, showed potent antifungal activity against both human and phytopathogenic fungi (52).

The technological steps used for beverages production, such as soaking, pressure cooking, and pasteurization, together with the discards of soaking and cooking waters, led to values of 7.12 and 7.65 mg/g for phytic acid in chickpea and lupin beverages, respectively, compared to higher phytic acid contents between 9.6 and 12.1 mg/g for cooked chickpea in other studies (53). This difference could be due to the combination of a long soaking time (16 h) and further leaching during the cooking step, which may have led to a more effective reduction of the final phytic acid, and the contents of other anti-nutritional factors in beverages.

Several differences between the minerals were observed in the available literature (54, 55). These variations may be due to different instruments used in the analysis (atomic absorption spectroscopy versus ICP-OES) along with pulse variety differences, edapho-climatic conditions, harvest period, and/or nutritional status of the plants that can influence the mineral contents of the seeds. On the contrary, different methods of *in vitro* digestion do not allow an accurate comparison between pulse mineral bioavailability and previous research. Despite that, the results of Zhang et al. (31) indicated that cooked sweet lupin seed was also a good source of K, Ca, Mg, and Mn, and all lupin mineral bioaccessibilities were higher than those obtained in this study. These results may also be explained by the possible leaching of minerals during beverage production, which can reduce their bioaccessibility (56).

The mineral contribution of 100 mL of pulse beverages for attaining the dietary reference intakes (DRI) established for adults (39, 57) (Table 4) was higher in lupin beverage for the majority of the mineral elements, except for copper in chickpea beverage, but all were lower than cow’s milk (58) and commercial vegetable beverages (59). This is supported by the fact that plant-based beverages usually show large variations in nutritional properties and a large discrepancy in nutrients and their bioavailability compared to milk (5, 50), leading to potential nutritional deficiencies if not well balanced through the food diet (60). In general, the composition of commercial vegetable beverages contains several additives, including minerals (especially calcium), to mimic the nutritional characteristics of cow’s milk (50). However, the objective of this study was to develop a technology to obtain a pulse-based beverage that incorporates the highest level of seed components (including husks), with no additives to meet the clean label concept.

Despite that, the pulse beverages obtained in this study could not have a nutrition claim for minerals, except for manganese (DRI > 7.5%) (39), which also showed bioaccessibility values of 2.94% and 5.64% for chickpea and lupin, respectively. This information is also essential for consumers to know the reliable nutritional content in a beverage (or food), as it should be considered not only the initial concentration of the nutrient to claim but also its bioaccessibility value after digestion. Thus, it could be right to consider that if lupin beverage (even without a “source of iron” claim) showed iron bioaccessibility of 31.43%, this should be taken into account for the beverage labeling, as it would be a good alternative for vegetarian consumers who usually present iron deficiencies (61). This discussion is increasingly relevant when we think about the meaning of a nutrient claim that, in our opinion, cannot be complete unless it is considered to be the bioaccessibility of the nutrient. In fact, the same *in vitro* method and mineral bioaccessibility evaluation of cow milk and soy milk should be used in further studies to compare accurately the differences between these two pulses-based beverages.

The *in vitro* method (29) used to mimic the human digestion of pulse-based beverages was successful in protein hydrolysis. Nevertheless, some polypeptides with 10 kDa (80–90 amino acid residues) were revealed in the whole *digesta*, and if soluble, these peptides are probably not absorbed by the small intestine. Those that are actively transported in intestinal epithelial cells are tripeptides, dipeptides, and free amino acids. However, the small intestinal digestion of proteins results in a mixture of oligopeptides dominated by dipeptides to hexapeptides (62). These oligopeptides are still hydrolyzed by brush border peptidase catalysis. The *in vitro* digestion model used in this study does not include brush border peptidases (29); therefore, it is not expected that pulse beverages should be completely broken down into free amino acids, dipeptides and tripeptides (63).

Results also showed that proteins in both beverages were well digested and that additionally, the few lectins present were mostly hydrolyzed, hence suggesting very good digestibility. Despite most lectins being resistant to degradation by digestive enzymes, lectin activities were clearly reduced in these beverages after digestion, showing that the cooking step used for beverage production was successful for protein inactivation and allowance for its nutritional increase (64).

Interestingly, the few low molecular weight proteins that seemed to remain unaffected through *in vitro* digestion presented similar molecular weight to the bioactive protein reported previously for chickpea and lupin, which contains high inhibitory bioactivities toward the gelatinases MMP-9 and MMP-2 (23, 24), particularly in lupin, where a low molecular weight oligomer with strong MMP-9 inhibitory activity was found to survive proteolysis during digestion and is effective in reducing colitis *in vivo* (24). If these bioactivities are kept, they can add much greater value to our beverages.

Indeed, our results concerning the bioactivities of both beverages (Table 5) show that both lupin and chickpea-based beverages do present a strong inhibitory activity on MMP-9 while reducing cancer cell migration. Similar results have been reported (23), where protein extracts from chickpea and lupin reduced MMP-9 activity and cancer cell migration but did not exert cellular toxicity; hence, cell proliferation remained essentially unaltered. It is important to note that, although previous reports have shown the presence of effective low molecular weight MMP-9 inhibitors in their protein fractions (23), these activities were found in raw and cooked protein extracts obtained from the seeds of these legumes. The fact that both chickpea and lupin beverages kept the MMP-9 inhibitory activity of their seeds after all the technological procedures and was even more pronounced (possibly due to a concentration effect) after *in vitro* digestion (Figure 3) suggests a very strong potential for digestive ailments related to MMP-9 impaired activities, such as IBD and colorectal cancer. The resistance to digestive enzymes could be related to the fact that these proteins are PIs, but nonetheless, this is an important fact to consider because most bioactive compounds (particularly concerning inflammatory diseases of the gastrointestinal tract) are inefficient because they are degraded throughout digestion and lose their activity. Hence, the fact that these bioactive polypeptides resist digestion and still carry out their bioactivities after all the digestive process is of great potential. This is in fact consistent with previous research that showed that the isolated *Lupinus* MMP-9 inhibitor is effective in reducing inflammation and lesions in animal models of induced colitis (24, 27) and cancer development (28). Hence, overall, our results seem to suggest that, in our pulse-based beverages, we were able to maintain these bioactive proteins; besides their nutrition value and digestibility, they also present high-added-value as functional foods to be used in preventive diets against colitis and IBDs.

## Conclusion

The simple technological process used in this study allowed the formulation of palatable and nutritionally rich pulse-based beverages, which can offer a sustainable alternative (okra-like residue lower than 2%) to dairy protein intolerance. The possible nutritional claims that could be used for both pulse beverages are as follows: “with no added sugar,” “fat-free,” “very low sodium,” and “source of manganese,” but for lupin beverage itself, it is possible to have one more specific allegation as “source of protein” (39), taking into account its protein content. The results also indicated that both pulse beverages are completely digested as soluble peptides that are available for further absorption. Future studies could be considered to study the bioavailability of the protein fractions with low-molecular weights.

Furthermore, the studied anti-nutritional compounds (phytic acid and lectins), naturally present in pulse seeds, are highly reduced through beverage processing and did not hinder minerals bioavailability and nonexistence of intestinal malabsorption events.

However, the mineral content of these pulse beverages, especially in calcium, magnesium, and phosphorus, is much lower compared to milk as mentioned above, and to obtain a sustainable vegetable alternative to milk, using a clean label approach, algae could be added to increase its mineral content. Several studies have been carried out and confirm this approach: Fradinho et al.’s study (65) showed a substantial increase in Ca, K, Mg, and I in gluten-free pasta enriched with *Laminaria ochroleuca* seaweed, and another study (66) increased more than 100% the calcium and iron content in gluten-free bread with microalgae addition. In the latter case, regardless of the strain used, 1% w/w of microalgae addition was sufficient to contribute over 15% of the recommended daily iron value (67).

In addition to being highly digestible and nutritious, lupin and chickpea beverages showed specific bioactivities of MMP-9 inhibition, as well as a reduction in the migration of colon cancer cells. Furthermore, the MMP-9 inhibitory activity was resistant to the digestion process, and is significantly enhanced, thus suggesting a strong potential as a functional food, for effective preventive diets against inflammatory and cancer diseases, especially those related to the digestive system.

## Data Availability Statement

The original contributions presented in this study are included in the article/supplementary material, further inquiries can be directed to the corresponding author.

## Author Contributions

IS, ARa, and RF: conceptualization. IS, ARa, AL, and ARi: methodology. CD, MN, ARi, JM, and AL: validation. CD, JM, AL, and MN: formal analysis. CD, JM, RA, CM, and ARi: investigation. IS, RF, RA, and CM: resources. CD and AL: writing—original draft preparation. CD, ARi, AL, IS, RF, RA, CM, ARa, and MN: writing—review and editing. CD and AL: visualization. IS and RF: supervision. IS: project administration and funding acquisition. All authors have read and agreed to the published version of the manuscript.

## Conflict of Interest

The authors declare that the research was conducted in the absence of any commercial or financial relationships that could be construed as a potential conflict of interest.

## Publisher’s Note

All claims expressed in this article are solely those of the authors and do not necessarily represent those of their affiliated organizations, or those of the publisher, the editors and the reviewers. Any product that may be evaluated in this article, or claim that may be made by its manufacturer, is not guaranteed or endorsed by the publisher.
